# A nonparametric test for equality of survival medians using right-censored prevalent cohort survival data

**DOI:** 10.1177/09622802221125912

**Published:** 2022-09-21

**Authors:** James Hugh McVittie, Masoud Asgharian

**Affiliations:** 177915 Department of Mathematics and Statistics, McGill University, Montreal, Canada

**Keywords:** length-bias, censoring, survival analysis, median

## Abstract

The median is a robust summary commonly used for comparison between populations. The existing literature falls short in testing for equality of survival medians when the collected data do not form representative samples from their respective target populations and are subject to right censoring. Such data commonly occur in prevalent cohort studies with follow-up. We consider a particular case where the disease under study is stable, that is, the incidence rate of the disease is stable. It is known that survival data collected on diseased cases, when the disease under study is stable, form a length-biased sample from the target population. We fill the gap for the particular case of length-biased right-censored survival data by proposing a large-sample test using the nonparametric maximum likelihood estimator of the survivor function in the target population. The small sample performance of the proposed test statistic is studied via simulation. We apply the proposed method to test for differences in survival medians of Alzheimer’s disease and dementia groups using the survival data collected as part of the Canadian Study of Health and Aging.

## 1 Introduction

In studies examining a survival time outcome for multiple groups/strata, it is typically of interest to the researcher to compare quantities related to the survival distribution such as the median. For example, Rahbar et al. compared the median waiting times for trauma patients to receive red blood cells using data from the Prospective Observational Multi-Center Major Trauma Transfusion study and, Brookmeyer and Crowley compared the median survival times for subjects receiving four types of cancer treatment using data from a Phase III colorectal cancer clinical trial.^1,2^ Hypothesis testing procedures based on comparing the median survival times between groups, for a variety of data types, have been well studied in the literature. For right-censored failure time data, Brookmeyer and Crowley proposed a testing procedure for comparing k sample medians using the asymptotic properties of the Kaplan–Meier product-limit estimator.^1,3^ Although their procedure was based on comparing median point estimates, they implicitly assumed that the failure time distributions are members of the same location family. Brookmeyer and Crowley defined a weighted Kaplan–Meier estimate to determine the sample median, however, the non-parametric estimate assumes the group survival distributions are equivalent. Given that members of a location family can be identified by their medians, these authors essentially test the equality of the underlying survival distributions. Rahbar et al. highlight this testing dichotomy by explaining that equality of distributions implies equality of medians whereas the converse statement is not necessarily true.^4^ Reid validated the bootstrapping procedure of Efron applied to the sample median for right-censored data and discussed the associated confidence interval properties.^5,6^

Recently, Rahbar et al. proposed a hypothesis testing procedure for comparing population medians using right-censored failure time data which does not require the location family assumption.^4^ Using the asymptotic distribution of the Kaplan–Meier estimator and an application of the functional delta method, Rahbar et al. derived a quadratic test statistic with an asymptotically chi-squared distribution.^7^ In this article, we propose an extension to the method of Rahbar et al. for the case in which the failure time data are both length-biased and right-censored using the nonparametric maximum likelihood estimator (NPMLE) of the unbiased survival function.^8^ We note that when the observed failure times are length-biased, the censoring mechanism is informative yielding an NPMLE which places mass on both the observed failure/censoring times of the prevalent cohort study.^8–11^ Thus, the unbiased survival function estimator is fully defined over the entire real line establishing the existence of the median point estimate. While other product-limit estimators for left-truncated right-censored failure time data may be applied (see, e.g. Huang and Qin,^12^ Luo and Tsai,^13^ and Tsai et al.^14^), the survival function estimates may not yield a median point estimate when censoring is high. Although there may be instances in which the median point estimate exists when utilizing the bootstrapping procedure of Efron, some bootstrap samples may not yield median estimates requiring them to be discarded.^5^ In addition to ensuring the existence of the median point estimate, our use of the NPMLE of the unbiased survival function is motivated by its efficiency compared to all other nonparametric estimators.^9^

In Section 2., we define the notation for prevalent cohort studies with follow-up resulting in length-biased right-censored failure time data and describe how the asymptotic representation of the NPMLE of the unbiased survival function may be utilized to define a statistic for testing differences between multiple population medians. In Section 3., we generate simulated failure time data from prevalent cohort studies with follow-up to assess the performance of the proposed testing procedure and in Section 4., we apply our testing methodology to the Canadian Study of Health and Aging (CSHA) dataset to compare median survival between various dementia/Alzheimer’s disease groups. We provide some extensions and future research directions in Section 5.

## 2 Notation and methodology

In a general prevalent cohort study with follow-up, subjects are screened and only diseased cases are followed until failure/censoring for which their respective onset dates are recorded retrospectively.^15^ Let the fixed constant R denote the date at which subjects are tested for the prevalence of the condition/disease under study (prevalence day) and let O<R denote a generic onset time generated from a stationary Poisson point process. Let T be the underlying failure random variable with distribution function $F_{U}(\cdot )=1-S_{U}(\cdot )$ and mean μ . A subject is included in the prevalent cohort study if T>R−O=A (i.e. they survive past prevalence day) where they are subsequently followed until the end of the study or they are lost to follow-up. Let C denote the censoring time measured from R with distribution function G(⋅) . Thus, the prevalent cohort consists of the triples $\left\{(A_{i},Y_{i},\delta_{i})=(R-O_{i},\min(T_{i},C_{i}),1_{\left\{T_{i}\leq C_{i}\right\}})\;:\;i=1,2,\ldots ,n\right\}$ made up of the observed truncation times, failure/censoring times and associated indicator functions. When the additional stationarity assumption is made for the underlying onset process of the prevalent cohort data (i.e. the underlying onset dates are drawn from a stationary Poisson point process) the observed failure times are typically referred to as being “length-biased.” For details on testing the validity of the stationary onset process assumption, see the literature.^16–19^ Let $F_{LB}(\cdot )=1-S_{LB}(\cdot )$ denote the length-biased distribution function for which it may be shown^8^:

(1) $$S_{U}(y)=\frac{\int_{y}^{\infty }\frac{1}{x}dS_{LB}(x)}{\int_{0}^{\infty }\frac{1}{x}dS_{LB}(x)};y>0$$

In this setting, it is sufficient to only use the observed failure/censoring times without the observed truncation times in order to make inferences regarding the unbiased survival function. For a set of length-biased right-censored failure time data, Asgharian and Wolfson established uniform consistency and weak convergence for the NPMLE of ${\hat{S}}_{LB}$ and showed

$$\sqrt{n}({\hat{S}}_{LB}-S_{LB})\overset{D}{\to }U=F^{-1}(V)$$

where U is a Gaussian process.^9^ Using the relationship between the unbiased and length-biased survival functions given in equation (1), Asgharian et al. established uniform consistency and weak convergence for the NPMLE of the unbiased survival function ${\hat{S}}_{U}$ .^8^ In particular, they established the following weak convergence result:

$$\sqrt{n}({\hat{S}}_{U}(y)-S_{U}(y))\overset{D}{\to }U^{*}(y)=\mu \int_{0}^{\infty }L_{y}(x)dU(x)$$

where $L_{y}(x)=I_{\left[y,\infty \right]}(x)-S_{U}(y)/x$ . As the operator acting on the limiting Gaussian process of ${\hat{S}}_{LB}$ is a bounded linear operator, it may then be shown that $U^{*}(y)$ is also a Gaussian process.^20^ ${}^{\left(p.\;377\right)}$

Using similar notation as Rahbar et al., consider the setting in which k≥2 independent populations are sampled for which the observed samples consist of length-biased failure/censoring times.^4^ We utilize the subscript j , j=1,…,k , to differentiate the parameters/estimators between the k populations/samples, respectively. For j=1,2,…,k , we define the median as:

(2) $$\theta_{U,j}=\inf\{t\;:\;S_{U,\;j}(t)\leq 0.5\}$$

for which the sample median estimator, ${\hat{\theta }}_{U,j}$ is determined via plugging in the estimator of ${\hat{S}}_{U,j}$ in equation (2).^4^ We note that the median estimators for the different groups will always exist as the corresponding unbiased survival function estimators, ${\hat{S}}_{U,j}$ , under the stationarity assumption, are fully defined over the entire real line. This is a direct result of the informative censoring mechanism which forces non-zero probability mass to fall on the observed censoring times.^21^ Moreover, as the observed length-biased data are collected from a prevalent cohort study with follow-up through a cross-section at prevalence day, the subjects’ onset dates may occur any time arbitrarily far before prevalence day. This implies that if the follow-up period is short, resulting in a large proportion of the observed failure times being right-censored, the NPMLE of the $S_{U,j}$ functions will be unaffected. For details on the NPMLE of $S_{U,j}$ without the stationarity assumption, see Tsai et al.^14^ For group j , j=1,2,…,k , it may be shown that the sample median estimator converges to a Gaussian random variable:

(3) $$\sqrt{n_{j}}\left\{{\hat{\theta }}_{U,j}-\theta_{U,j}\right\}\to Z_{\;j}∼N(0,\sigma_{j}^{2})$$

We summarize the necessary conditions of this result with a sketch of its proof in Theorem 1 and Corollary 4 in the Appendix. Estimates for $\sigma_{j}^{2}$ , j=1,2,…,k may be obtained by applying the simple bootstrap procedure of Efron to each of the k samples by resampling the pairs $\left(Y_{i},\delta_{i}\right)$ , $i=1,2,\ldots ,n_{\;j}$ , j=1,2,…,k .^5^

Let $\theta_{U,1},\ldots ,\theta_{U,k}$ be the population medians and let $n_{1},\ldots ,n_{k}$ be the sample sizes for each of the k samples. Consider the null hypothesis of equal medians for each of the k groups:

$$H_{0}\;:\;\theta_{U,1}=\cdots =\theta_{U,k}(=\theta_{0})$$

Following the approach of Rahbar et al., we define the combined median estimator as:

$${\hat{\theta }}^{c}=({\hat{\theta }}_{N}^{c},\ldots ,{\hat{\theta }}_{N}^{c})\;\mathrm{where}\;{\hat{\theta }}_{N}^{c}=\sum_{\;j=1}^{k}\frac{n_{j}/{\hat{\sigma }}_{j}^{2}}{\left(n_{1}/{\hat{\sigma }}_{1}^{2})+\cdots +(n_{k}/{\hat{\sigma }}_{k}^{2}\right)}{\hat{\theta }}_{j}$$

which can be re-expressed as

$${\hat{\theta }}^{c}=\sum_{\;j=1}^{k}{\hat{L}}_{j}{\hat{\theta }}_{j}$$

where

$${\hat{L}}_{j}=\frac{n_{j}/{\hat{\sigma }}_{j}^{2}}{\left(n_{1}/{\hat{\sigma }}_{1}^{2})+\cdots +(n_{k}/{\hat{\sigma }}_{k}^{2}\right)}$$

for which $\sum_{\;j=1}^{k}{\hat{L}}_{j}=1$ .^4^ Denoting the combined sample size $N=n_{1}+\cdots +n_{k}$ , Rahbar et al. proposed the following quadratic test statistic:

(4) $${\hat{\Lambda }}_{N}=N(\hat{\theta }-{\hat{\theta }}^{c}{)}^{\prime }{\hat{\Gamma }}_{N}^{-1}(\hat{\theta }-{\hat{\theta }}^{c})$$

where ${\hat{\Gamma }}_{N}$ is a k×k matrix with diagonal entries given by

$${\hat{\Gamma }}_{Ni}={\hat{\sigma }}_{i}^{2}w_{i,N}^{-1}(1-{\hat{L}}_{i}{)}^{2}+\sum_{\;j\neq i}^{k}{\hat{\sigma }}_{j}^{2}w_{\;j,N}^{-1}{\hat{L}}_{\;j}^{2};i=1,2,\ldots ,k$$

and off-diagonal entries given by

$${\hat{\Gamma }}_{Nij}=-{\hat{L}}_{i}w_{i,N}^{-1}{\hat{\sigma }}_{i}^{2}-{\hat{L}}_{j}w_{\;j,N}^{-1}{\hat{\sigma }}_{j}^{2}+\sum_{m=1}^{k}{\hat{\sigma }}_{m}^{2}w_{m,N}^{-1}{\hat{L}}_{m}^{2};i\neq j=1,2,\ldots ,k$$

for which $w_{i,N}=n_{i}/N$ for i=1,2,…,k .^4^ As remarked by Rahbar et al., the matrix $\Gamma_{N}$ is not necessarily invertible and so the generalized inverse may be utilized instead of expression (4). Using the asymptotic normality of the sample median estimators, ${\hat{\theta }}_{U,j}$ , j=1,2,…,k , $\Lambda_{N}$ may be shown to weakly converge to a central $\chi^{2}$ distribution with k−1 degrees of freedom (see Theorem 2 of Rahbar et al.^4^). The percentiles of the central $\chi^{2}$ distribution may be utilized to test for significance when conducting the equality of medians hypothesis test.

### 2.1 Length-biased/unbiased survival function median comparisons

**Figure 1.**
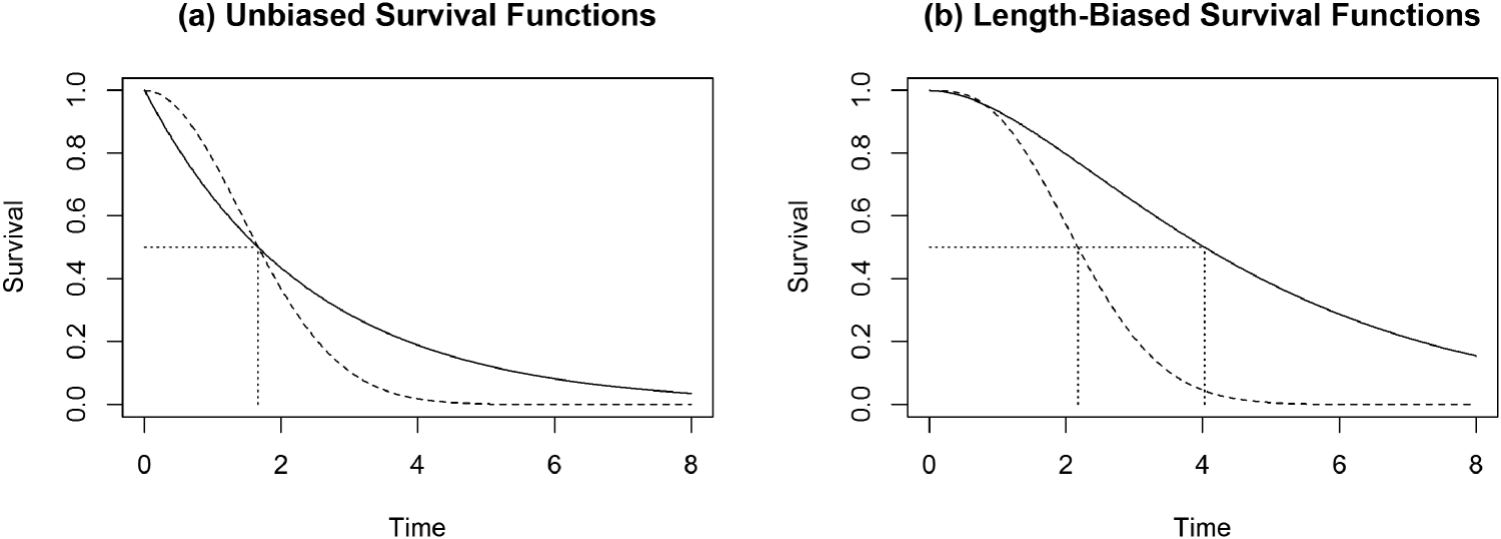
Illustration comparing unbiased and length-biased survival curves for two groups. The two unbiased survival curves were generated from Weibull distributions with (shape, scale) parameters of (2,2) and $\left(1,2\log\;(2{)}^{\left(1/2\right)}/\log\;(2)\right)$ yielding equal unbiased medians indicated by the single vertical line in panel (a). The length-biased medians, computed using the length-biased survival functions, are indicated by the two distinct vertical lines in panel (b).

Although the proposed testing procedure is based on determining differences between the unbiased survival function medians, a question that may be posed is whether the correct conclusion will be reached when testing for equality of the length-biased population medians instead. Using the weak convergence of the length-biased survival function NPMLE ${\hat{S}}_{LB}(\cdot )$ , an equivalent form of the test statistic in equation (4) may be formed to test the null hypothesis:

$$H_{0}\;:\;\theta_{LB,1}=\theta_{LB,2}=\cdots =\theta_{LB,k}(=\theta_{0}^{\prime })$$

where $\theta_{LB,j},j=1,2,\ldots ,k$ are the population medians of the length-biased survival functions. For simplicity of exposition, consider the case with k=2 in which the medians of two survival distributions are being compared. Addona examined different types of group comparisons between unbiased and length-biased survival curves and found that in the two-sample setting, there exist examples in which $\theta_{LB,1}<\theta_{LB,2}$ , however, $\theta_{U,1}>\theta_{U,2}$ (i.e. the ordering of medians is “reversed”).^22^ In Figure 1, we illustrate a similar phenomenon using Weibull failure time data and show that the unbiased survival curves for two groups can have identical medians whereas the medians of the corresponding length-biased survival curves are clearly different. Therefore, without restriction on the family of underlying failure time distributions, it is not sufficient to test for differences in medians of the length-biased distributions and then subsequently make conclusions regarding the unbiased population medians. For related discussions on testing for equality of survival curves using length-biased failure time and adjusting for time-dependent selection mechanism/covariates, see Ning et al.^10^ and Huiart and Sylvestre.^23^

## 3 Simulations

To assess the performance of the proposed hypothesis test, we generated samples of length-biased right-censored failure time data from various distributions to control for the values of the unbiased population medians. Specifically, we considered three failure time distributions: uniform distribution with support (0,2θ) (median equal to θ ), the LogNormal distribution with parameters μ and 1 (median equal to $e^{\mu }$ ) and the exponential distribution with rate parameter log(2)/λ (median equal to λ ). To generate a sample of length-biased right-censored failure time data, we sampled an onset time, O , from a uniform distribution over (0,R) where R was chosen arbitrarily large such that P(T>R)≈0 . We then sampled a failure time T and retained both the onset, failure time pair if T>R−O , otherwise both sampled quantities were discarded. This procedure of accepting/rejecting the sampled (onset, failure time) pairs simulates the effect of left-truncation on the underlying failure time random variables. We censored the forward failure times T−(R−O) by drawing an exponentially distributed censoring time, C , with rate parameter ϕ where we recorded min(C,T−(R−O)) and whether the minimum was a failure/censoring time. The rate parameter ϕ was varied to allow for either 15% or 30% censoring.

We generated multiple length-biased right-censored failure time data sets and computed the empirical test sizes and empirical powers when the medians of the distributions were equal and different, respectively. We considered the case in which k=3 where we allowed the underlying failure time distributions to be equal or different from each other. We set the sample sizes of the three groups equal to 150 , 200 , and 250 , respectively, and performed the same simulations when all three samples were of size 50 . We ran 500 simulation runs and for each run, we estimated the sample median variances using 1000 bootstrapped samples. We reported the empirical test sizes in Table 1 and the empirical powers in Table 2. We find that for the majority of cases when the sample sizes are large, the empirical test sizes are similar to the true test significance level of α=0.05 . When the sample sizes of the three groups were small, the empirical sizes increased in most cases. In the cases involving the exponential distribution, we attribute the larger empirical test sizes to the difficulty in drawing a sufficient number of observations arbitrarily close to time t=0 . In Table 2, we find that when the sample sizes are large, the empirical powers are in most cases above 0.50 and when the sample sizes are small, the empirical powers are diminished. This phenomenon is to be expected as with smaller sizes, the variability of the estimators’ increases and it becomes more challenging to detect any differences between the underlying population medians.

**Table 1.** Empirical test sizes for simulated length-biased right-censored failure times of three groups drawn from underlying survival distributions with equal medians of θ=10 . Large sample sizes of three groups: $n_{1}=150$ , $n_{2}=200$ , $n_{3}=250$ . Small sample sizes of three groups: $n_{1}=n_{2}=n_{3}=50$ . Simulation runs: 500. Number of bootstrapped samples per run: 1000.

	Large samples		Small samples	
	Censoring percentage			
Group distribution types	15%	30%	15%	30%
All Uniform	0.050	0.040	0.110	0.088
All LogNormal	0.060	0.052	0.066	0.064
All Exponential	0.100	0.100	0.122	0.116
2 Uniform, 1 LogNormal	0.056	0.048	0.106	0.096
2 Uniform, 1 Exponential	0.080	0.070	0.120	0.108
2 LogNormal, 1 Uniform	0.048	0.058	0.066	0.076
2 LogNormal, 1 Exponential	0.084	0.086	0.088	0.082
2 Exponential, 1 Uniform	0.086	0.078	0.134	0.126
2 Exponential, 1 LogNormal	0.086	0.082	0.126	0.112
1 Uniform, 1 LogNormal, 1 Exponential	0.084	0.082	0.106	0.104

**Table 2.** Empirical powers of a hypothesis test for simulated length-biased right-censored failure times of three groups drawn from underlying survival distributions with varying medians. Large sample sizes of three groups: $n_{1}=150$ , $n_{2}=200$ , and $n_{3}=250$ . Small sample sizes of three groups: $n_{1}=n_{2}=n_{3}=50$ . Simulation runs: 500. Number of bootstrapped samples per run: 1000.

	Large samples		Small samples	
	Censoring percentage			
Group distribution types and group medians $\left(\theta_{1},\theta_{2},\theta_{3}\right)$	15%	30%	15%	30%
All Uniform (10,10,6)	0.728	0.690	0.718	0.656
All LogNormal $\left(10,10,e^{2}\right)$	0.364	0.368	0.102	0.110
All Exponential (10,10,20)	0.690	0.674	0.342	0.314
2 Uniform, 1 LogNormal $\left(10,10,e^{2}\right)$	0.544	0.518	0.220	0.200
2 Uniform, 1 Exponential (10,10,20)	0.742	0.732	0.354	0.338
2 LogNormal, 1 Uniform (10,10,6)	0.660	0.588	0.636	0.560
2 LogNormal, 1 Exponential (10,10,20)	0.736	0.726	0.352	0.306
2 Exponential, 1 Uniform (10,10,6)	0.664	0.620	0.658	0.598
2 Exponential, 1 LogNormal $\left(10,10,e^{2}\right)$	0.428	0.408	0.212	0.210
1 Uniform, 1 LogNormal, 1 Exponential $\left(10,e^{2},20\right)$	0.896	0.896	0.496	0.462

**Figure 2.**
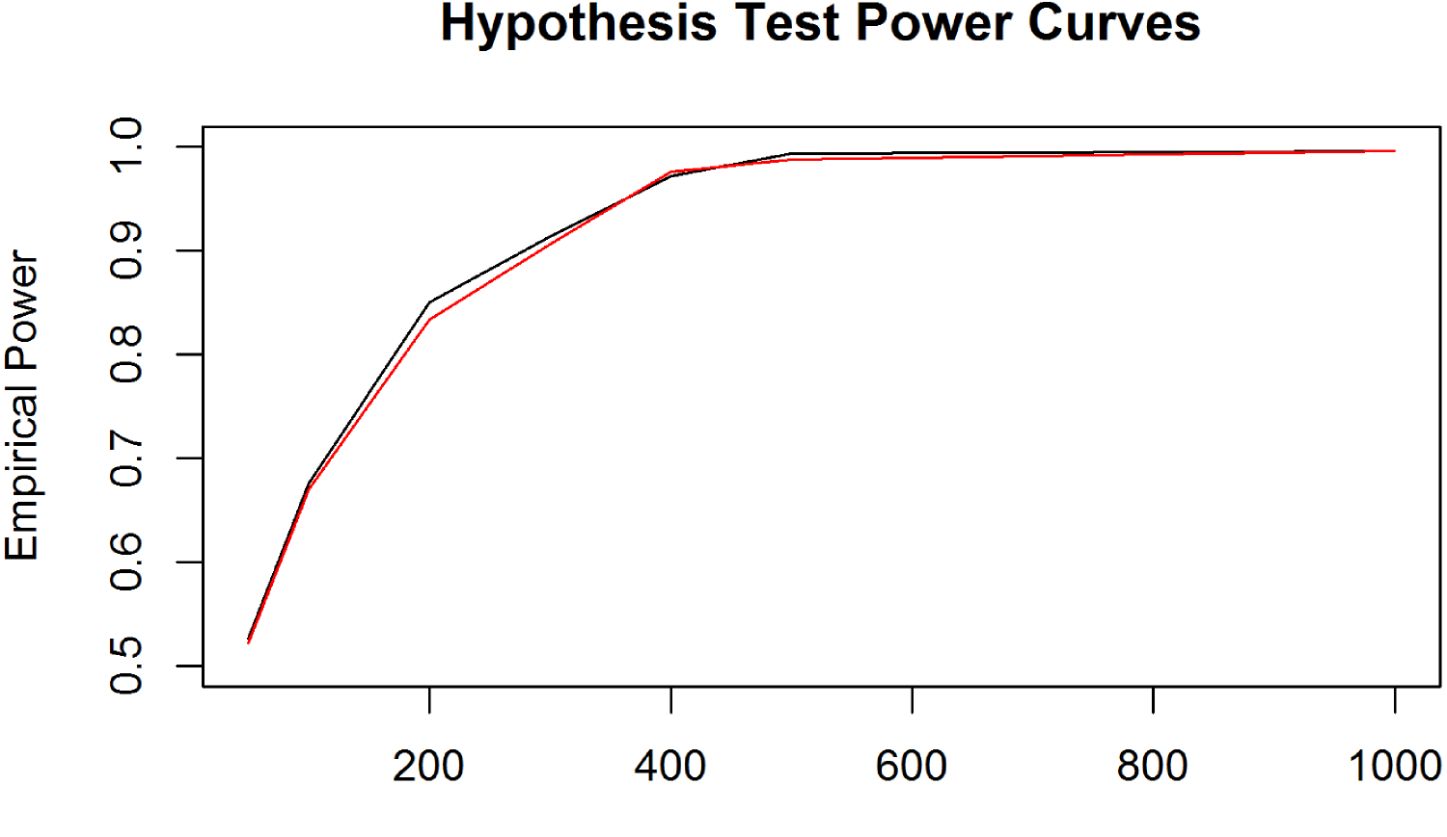
Empirical power curves of the median comparison hypothesis test for simulated length-biased right-censored failure data of three groups drawn from Uniform, LogNormal, and Exponential distributions with respective medians of 10 , $e^{2}$ , and 20 . The empirical powers were computed when the sample sizes of the three groups $n_{1}=n_{2}=n_{3}$ were equal to 50 , 100 , 200 , 300 , 400 , 500 , and 1000 , where the proportion of censoring in the three groups was equal to 15% (black) or 30% (red). Simulation runs: 500 . Number of bootstrapped samples per run: 1000 .

To assess how the power of the hypothesis test varied depending on the group sample sizes, we simulated length-biased right-censored failure time data from three groups with different medians (Uniform: 10, LogNormal: $e^{2}$ , Exponential: 20). We varied the censoring variable’s rate parameter, ϕ , so that either 15% or 30% of the sampled length-biased failure times were right-censored. We computed the empirical powers over 500 simulation runs with 1000 bootstrapped samples per run where all three sample sizes were equal to 50 , 100 , 200 , 300 , 400 , 500 , and 1000 . The empirical power curves are plotted in Figure 2. Generally, as the sample size of the individual groups’ increases, the power of the test converges to 1 independently of the two different proportions of censoring between the three groups.

## 4 Application

The CSHA was a multicenter study of dementia and related geriatric conditions for individuals over the age of 65 in Canada.^24^ In 1991, in the first stage of the CSHA, approximately 10,000 subjects living in a community or institutional residences were screened for various forms of cognitive impairment. At the second stage of the CSHA in 1996, the subjects who were still alive were screened a second time for cognitive impairment through a clinical evaluation. For the subjects who screened positive at the first stage of the CSHA, their onset dates were obtained through the recollections of their family members and caregivers and the dates of death were recorded for those subjects who died between the first and second stages of the CSHA. Through the screening tests and the clinical evaluations, the prevalent cohort of subjects ( n=823 ) consisting of 240 males and 583 females who screened positive at the first stage of the CSHA were classified as having either vascular dementia ( $n_{1}=173$ ), possible Alzheimer’s disease ( $n_{2}=252$ ) or probable Alzheimer’s disease ( $n_{3}=396$ ). Subjects’ survival times were (administratively) right-censored if they were still alive at the time of the second stage of the CSHA. Approximately 19% , 24% , and 21% of the subjects screened with vascular dementia, possible Alzheimer’s disease, or probable Alzheimer’s disease, respectively, were right-censored at the second stage of the CSHA.

**Figure 3.**
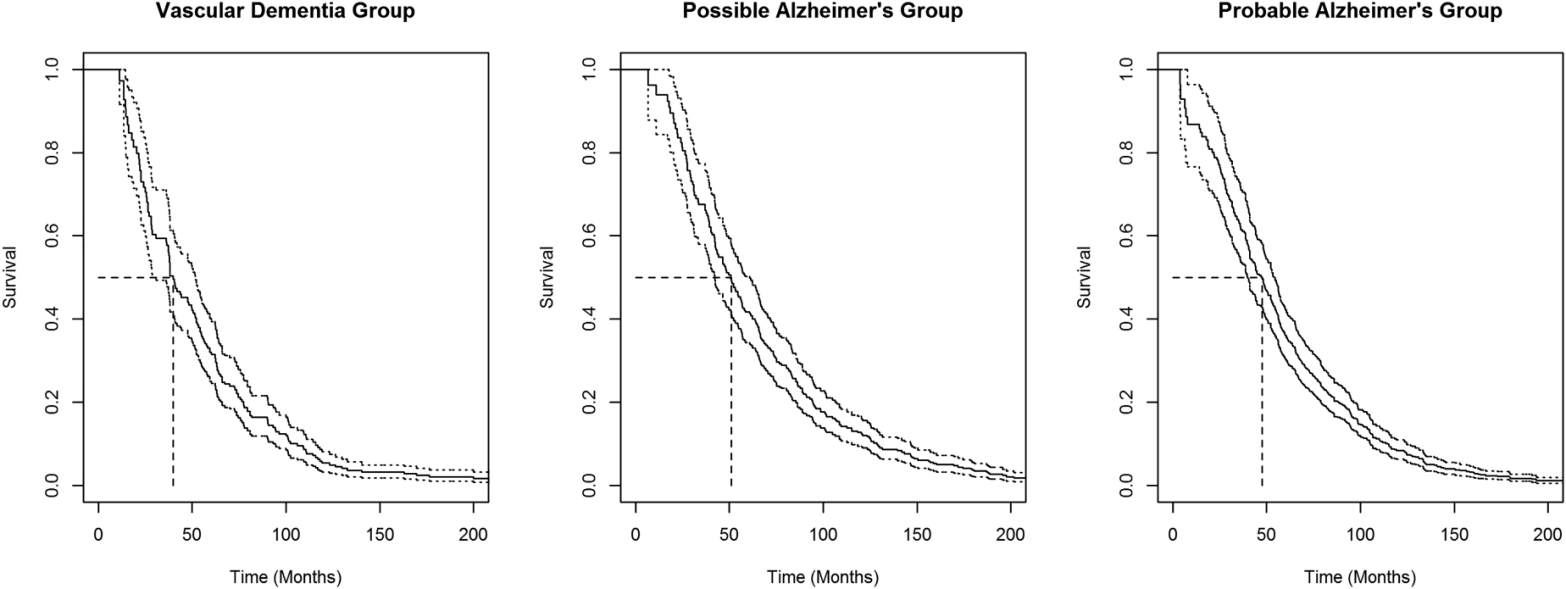
Nonparametric maximum likelihood survival function estimates for vascular dementia, possible Alzheimer’s disease, and probable Alzheimer’s disease groups in the Canadian Study of Health and Aging. Corresponding pointwise 95% confidence intervals are indicated with dotted lines and the median estimates for each group are indicated by dashed lines.

Using the observed failure/censoring times for the three dementia/Alzheimer’s disease groups of the CSHA, we calculated the unbiased survival function estimates and the associated unbiased survival function median estimates (see Figure 3). The medians for vascular dementia, possible Alzheimer’s disease, and probable Alzheimer’s disease groups (in months) were 39.9 , 50.9 , and 47.7 , respectively. For other median estimates and estimates of the hazard ratio for death, stratifying by sex, education level, and age at onset of dementia, see Wolfson et al.^24^ Using the simple bootstrap, we generated 10,000 bootstrap samples for each group and determined estimates of the sample median variances. Setting the significance level α=0.05 , using the bootstrapped estimates, we calculated the test statistic from equation (4), $\Lambda_{n}=2.988$ , and did not reject the null hypothesis that individual dementia/Alzheimer’s disease group medians are equal.

## 5 Discussion

The median estimator is a key summary statistic for determining the center of a failure time distribution and can be utilized to compare the failure time distributions of multiple independent groups. Using length-biased right-censored failure time data collected from a prevalent cohort study with follow-up, we proposed an extension to the Rahbar et al. testing procedure using the NPMLE of the unbiased survival function to compare the unbiased medians of k≥2 groups/populations. We applied our testing procedure to simulated prevalent cohort data sets and to data collected from the CSHA.

Throughout this article, we proposed a testing procedure for comparing the medians of multiple groups/populations using length-biased right-censored failure time; however, a closely related function to the median survival time is the quantile residual lifetime function (qrlt function). The qrlt function $\theta_{\alpha }(t)$ satisfies the equality $P(T-t\geq \theta_{\alpha }(t)|T\geq t)=\alpha$ for all t≥0 and some α∈[0,1] and determines the conditional α quantile when T≥t . The median survival time may be regarded as a single point of the qrlt function when α=0.5 and t=0 . Recently, Liu et al. utilized the asymptotic properties of the unbiased survival function NPMLE to develop confidence intervals for two test statistics based on the ratio and difference of two qrlt functions.^11^ It is not immediately clear how to utilize the proposed test statistics in practice when performing a hypothesis test of equality of qltr functions. It remains an open problem how the proposed methodologies may be extended to the k>2 sample setting in which multiple population qrlt functions are being compared.

## Appendix: Theoretical notes

To establish weak convergence of the median estimator, we consider the median residual lifetime function, $\theta_{t}$ , where the function $\theta_{t}$ must satisfy the relation given by

$$P(T>t+\theta_{t}|T>t)=\frac{1}{2}$$

which in terms of the unbiased survival function is expressed as

$$S_{U}(t+\theta_{t})=\frac{1}{2}S_{U}(t).$$

Observe the median, $\theta_{U}$ , may be regarded as a special instance of $\theta_{t}$ , where t=0 . The above expression yields the following estimating equation for $\theta_{t}$ :

(5)
$$u(\theta_{t})=S_{U}(t+\theta_{t})-\frac{1}{2}S_{U}(t)$$

where the plug-in estimator is given by

(6)
$$\hat{u}(\theta_{t})={\hat{S}}_{U}(t+\theta_{t})-\frac{1}{2}{\hat{S}}_{U}(t)$$

and the median residual lifetime function estimator, ${\hat{\theta }}_{t}$ , is defined as the root of equation (6). Using the results of Asgharian and Wolfson,^9^ some reasonable assumptions on $f_{U}(x)=-(d/dx)S_{U}(x)$ and a weak assumption on the behaviour of $S_{U}(\cdot )$ for small failure times, we show the estimating equation $\hat{u}(\theta_{t})$ is consistent and is weakly convergent to a process in, $D_{0}[0,\infty )$ , the space of cadlag functions over [0,∞) , endowed with the uniform topology, the topology induced by the uniform norm. We show that the estimator ${\hat{\theta }}_{t}$ is also consistent as well as weakly convergent using results from the theory of Z-estimators.^20^

Theorem 1.
Define $\tau =\inf\{t\;:\;F_{LB}(t)=1\}$ . Let τ<∞ . Suppose ∃γ such that $S_{U}(y)=1$ for y<γ with $\mu =\int_{0}^{\infty }xdF_{U}(x),$ where $F_{U}(\cdot )=1-S_{U}(\cdot )$ and $f_{U}$ has a bounded derivative. Let $S_{C^{\prime }}(\cdot )$ denote the survival function of the residual censoring variable, $\alpha (t)=(1/t)\int_{0}^{t}S_{C^{\prime }}(u)du$ and $\beta (t)=1-S_{C^{\prime }}(t)$ . Suppose for τ<∞ , ([2/α(τ)]−1)β(τ)<1 . Then the following statements hold:

1. $\underset{0\leq t<\infty }{\sup}|\hat{u}(\theta_{t})-u(\theta_{t})|\leq \frac{6\mu \|{\hat{S}}_{LB}-S_{LB}{\|}_{\infty }}{\gamma }$ .
2. $\underset{0\leq t<\infty }{\sup}|\hat{u}(\theta_{t})-u(\theta_{t})|=O(\sqrt{\frac{\log\;\log\;n}{n}})$ .
3. $\sqrt{n}(\hat{u}(\theta_{t})-u(\theta_{t}))\overset{D}{\to }\mu \int_{0}^{\infty }(L_{t+\theta_{t}}(x)-\frac{1}{2}L_{t}(x))dU(x)$ ,
  where $L_{y}(x)=\frac{1}{x}(I_{\left[y,\infty \right]}(x)-S_{U}(y))$ for x,y≥γ and 0 otherwise, and U , given by equation (8) in Asgharian and Wolfson,^9^ is a Gaussian process.
4. $\hat{u}(\theta_{t})$ is asymptotically efficient for estimating $u(\theta_{t})$ .
5. ${\hat{\theta }}_{t}\overset{P}{\to }\theta_{t}$ as n→∞ .
6. $\sqrt{n}f_{U}(t+\theta_{t})({\hat{\theta }}_{t}-\theta_{t})=\sqrt{n}\hat{u}(\theta_{t})+o_{P}(1)$ .

Definition 1.
A stochastic process X(t) is “cadlag” for every point $t_{0}\in R$ if the process X(⋅) is continuous at $t_{0}$ from the right with limit existing at $t_{0}$ from the left. Specifically, for every point $t_{0}\in R$ , for all sequences $\{t_{1n}{\}}_{n=1}^{\infty },$ which converge to $t_{0}$ from the right, $P(\lim_{n\to \infty }X(t_{1n})=X(t_{0}))=1$ and for all sequences $\{t_{2n}{\}}_{n=1}^{\infty },$ which converge to $t_{0}$ from the left, $P(\lim_{n\to \infty }X(t_{2n})=X(t_{0}-))=1$ .

Proposition 2. Let X(t) be a cadlag stochastic process and let g(t) be a continuous function ∀t∈R . The stochastic process X(g(t)) will also be a cadlag stochastic process.

Proof.
Let $t_{0}$ be an arbitrary point in R . Then since X(⋅) is a cadlag stochastic process, either

1. X is continuous at $t_{0}$ from both the left and right such that for all convergent sequences $\{t_{n}\in R\;:\;n=1,2,\ldots \},$ which converge to $t_{0}$ , we have that $P(\lim_{n\to \infty }X(t_{n})=X(t_{0}))=1$ , or
2. X has a limit, which exists at $t_{0}$ from the left and X is continuous at $t_{0}$ from the right such that for all convergent sequences $\{t_{n}\in R\;:\;n=1,2,\ldots \},$ which converge to $t_{0}$ from the left and for all convergent sequences $\{t_{n}^{*}\in R\;:\;n=1,2,\ldots \},$ which converge to $t_{0}$ from the right, we have that $P(\lim_{n\to \infty }X(t_{n})=X(t_{0}-))=1\text{\textbackslash{};}and\;P(\lim_{n\to \infty }X(t_{n}^{*})=X(t_{0}))=1$ .

Let $t_{n}$ be an arbitrary sequence, which converges to $t_{0}$ . Since g is a continuous function then $g(t_{n})\to g(t_{0})$ . Since g is continuous, there exists a small ball around $t_{0}$ of radius ϵ>0 such that for all n>N , for some N large enough, either $g(t_{n})\geq g(t_{0})$ , $g(t_{n})\leq g(t_{0})$ or neither of these cases (i.e. locally, the sequence $g(t_{n})$ alternates being smaller than or greater than $g(t_{0})$ ). If $g(t_{n})\geq g(t_{0})$ , then $g(t_{n})\to g(t_{0})$ from the right and since X is continuous from the right then $P(\lim_{n\to \infty }X(g(t_{n}))\to X(g(t_{0})))=1$ . If $g(t_{n})\leq g(t_{0})$ , then $g(t_{n})\to g(t_{0})$ from the left and since X has limits from the left then $P(\lim_{n\to \infty }X(g(t_{n}))\to X(g(t_{0})-))=1$ . If $g(t_{n})$ alternates from being greater than or less than, locally, $g(t_{0})$ , then if X is continuous from both the left and right at $g(t_{0})$ then $P(\lim_{n\to \infty }X(g(t_{n}))\to X(g(t_{0})))=1$ . Otherwise, we have that $P(X(g(t_{n}))\to X(g(t_{0})))=0,$ which implies that $X(g(t_{0}))$ is a point of discontinuity. If this is the case, take all convergent subsequences of $g(t_{n}),$ which approach $g(t_{0})$ from the right and all convergent subsequences of $g(t_{n}),$ which approach $g(t_{0})$ from the left and then apply the previous argument to show that X is cadlag at $g(t_{0})$ . Therefore, from the above arguments, we have shown that if g is a continuous function, then for any given $t_{0}\in R$ , that either $g(t_{0})$ is a point of class (a) or of class (b) thus proving that X(g(t)) is a cadlag stochastic process.  □

Lemma 3.
The function $\theta_{t}$ is continuous.

Proof.
Suppose the unbiased density function $f_{U}(x)$ is a continuous positive function on its support. For a fixed $t_{0}$ , we have the following equality:

$$\int_{t_{0}+\theta_{t_{0}}}^{\infty }f_{U}(x)dx=\frac{1}{2}\int_{t_{0}}^{\infty }f_{U}(x)dx.$$

Let $t\to t_{0}^{+}$ and note that since $\theta_{t}$ is a non-decreasing function, by definition, we have

$$\int_{t_{0}+\theta_{t_{0}}}^{t+\theta_{t}}f_{U}(x)dx=\frac{1}{2}\int_{t_{0}}^{t}f_{U}(x)dx$$

By applying the mean-value theorem, and taking the limit, we find that $\lim_{t\to t_{0}^{+}}\theta_{t}=\theta_{t_{0}}$ since f is a continuous positive function on its support. Through a similar argument, we can show left-continuity. Thus, $\theta_{t}$ is a continuous function.  □

Proof of Theorem 1.

1. From equation (A.1) of Asgharian et al.,^8^ we have
  $${\hat{S}}_{U}(y)-S_{U}(y)=(1-{\hat{S}}_{U}(y))\frac{\int_{0}^{\infty }d[{\hat{S}}_{LB}(x)-S_{LB}(x)]/x}{\int_{0}^{\infty }dS_{LB}(x)/x}-\frac{\int_{0}^{y}d[{\hat{S}}_{LB}(x)-S_{LB}(x)]/x}{\int_{0}^{\infty }dS_{LB}(x)/x}$$
  Using the triangle inequality, we have the following bound:
  $$\underset{0\leq \theta_{t_{0}}<\infty }{\sup}|\hat{u}(\theta_{t_{0}})-u(\theta_{t_{0}})|\leq \underset{0\leq \theta_{t_{0}}<\infty }{\sup}|{\hat{S}}_{U}(t_{0}+\theta_{0})-S_{U}(t_{0}+\theta_{t_{0}})|+\frac{1}{2}\underset{0\leq t_{0}<\infty }{\sup}|{\hat{S}}_{U}(t_{0})-S_{U}(t_{0})|$$
  Under the assumption that there exists γ>0 such that $S_{U}(y)=1,{\hat{S}}_{U}(y)=1$ for y<γ , then
  $$\begin{array}{rl} & \leq \underset{0\leq t_{0}+\theta_{t_{0}}<\infty }{\sup}|{\hat{S}}_{U}(t_{0}+\theta_{t_{0}})-S_{U}(t_{0}+\theta_{t_{0}})|+\frac{1}{2}\left(\frac{4\mu \|{\hat{S}}_{LB}-S_{LB}{\|}_{\infty }}{\gamma }\right)\\ & \leq \frac{4\mu \|{\hat{S}}_{LB}-S_{LB}{\|}_{\infty }}{\gamma }+\frac{2\mu \|{\hat{S}}_{LB}-S_{LB}{\|}_{\infty }}{\gamma }=\frac{6\mu \|{\hat{S}}_{LB}-S_{LB}{\|}_{\infty }}{\gamma } \end{array}$$
2. Under an assumption on the censoring distribution given by equation (6) in Asgharian et al.,^8^ the result of item 2 follows immediately.
3. We have that for a continuous mapping $\theta_{t}$ that the function $\Gamma (f)(t)=f(t+\theta_{t})-\frac{1}{2}f(t)$ is also continuous linear map. We also have from Proposition 2 that since ${\hat{S}}_{U}(\cdot )$ is a cadlag function, then ${\hat{S}}_{U}(t+\theta_{t})-\frac{1}{2}{\hat{S}}_{U}(t)$ is also a cadlag function. Given this setup, the result of item 3 follows from the results of Asgharian et al.^8^ and Asgharian and Wolfson.^9^
4. From Theorem 25.50 of van der Vaart,^25^ the pair $\left({\hat{S}}_{U}(t+\theta_{t}),{\hat{S}}_{U}(t)\right)$ is efficient for $\left(S_{U}(t+\theta_{t}),S_{U}(t)\right)$ . By applying the function $f(x,y)=x-\frac{1}{2}y$ to the pair of efficient estimators, since f is Hadamard differentiable, then by Theorem 25.47, the estimator $\hat{u}(\theta_{t})$ is efficient for $u(\theta_{t})$ .
5. By item 1 of Theorem 1, and from Asgharian et al.,^8^ $\underset{0\leq t<\infty }{\sup}|\hat{u}(\theta_{t})-u(\theta_{t})|\overset{P}{\to }0$ . Since the function $\theta_{t}$ is assumed to be identifiable uniformly for all t>0 , then by Theorem 5.9 of van der Vaart,^25^ any sequence of estimators, ${\hat{\theta }}_{t}$ which are roots to equation (6) is consistent for the true $\theta_{0,t}$ .
6. Let $\Gamma (\theta )(t)=u(\theta_{t})$ , and let $\lambda (t)={\hat{\theta }}_{t}-\theta_{t}$ then
  $$\Gamma (\theta +\lambda )(t)-\Gamma (\theta )(t)=S_{U}(t+\theta_{t}+\lambda (t))-S_{U}(t+\theta_{t}).$$
  Using the mean-value theorem and a second-order Taylor expansion, we obtain
  $$=S_{U}^{\prime }(t+\theta_{t})\lambda (t)+S_{U}^{\prime \prime }(\psi (t))\lambda^{2}(t)=-f_{U}(t+\theta_{t})\lambda (t)+o(\|\lambda \|)$$
  where the assumption of the boundedness of $S_{U}^{\prime \prime }$ guaranteed the last equality. Assuming the derivative has a continuous inverse and by applying the results of items 1–5, then by Theorem 19.26 of van der Vaart,^25^ we obtain the weak convergence result of item 6.

 □

As remarked above, the median point estimator may be regarded as the median residual lifetime estimator evaluated at t=0 , ${\hat{\theta }}_{0}={\hat{\theta }}_{U}$ . Using this relation, we state the following weak convergence result for ${\hat{\theta }}_{U}$ .

Corollary 4.
Let $\left\{(Y_{i},\delta_{i})=(\min(T_{i},C_{i}),1_{\left\{T_{i}\leq C_{i}\right\}})\;:\;i=1,2,\ldots ,n\right\}$ denote a sample of length-biased failure/censoring times and the associated indicator functions. Suppose conditions of Theorem 1 are fulfilled, then as n→∞

$$\sqrt{n}\left\{{\hat{\theta }}_{U}-\theta_{U}\right\}\overset{D}{\to }Z∼N(0,\sigma^{2})$$

where

$$\sigma^{2}={\left(\frac{\mu }{f_{U}(\theta_{U})}\right)}^{2}\int_{0}^{\tau }\int_{0}^{\tau }\psi (s,t)dL_{\theta_{U}}(t)dL_{\theta_{U}}(s)$$

and ψ(s,t)=cov(U(s),U(t)) .

Proof.
Follows immediately from Theorem 1, item 3, when t=0 .  □
